# Disparities in postpartum depression screening participation between immigrant and Danish-born women

**DOI:** 10.1093/eurpub/ckab197

**Published:** 2021-12-02

**Authors:** Maria Marti-Castaner, Camila Hvidtfeldt, Sarah Fredsted Villadsen, Bjarne Laursen, Trine Pagh Pedersen, Marie Norredam

**Affiliations:** 1 Department of Public Health, Section of Health Services Research, University of Copenhagen, Copenhagen, Denmark; 2 ROCKWOOL Foundation Research Unit, Copenhagen, Denmark; 3 Department of Public Health, Section of Social Medicine, University of Copenhagen, Copenhagen, Denmark; 4 Department of Health and Social Context, National Institute of Public Health, University of Southern Denmark, Copenhagen, Denmark

## Abstract

**Background:**

Qualitative studies suggest that immigrant women experience barriers for postpartum depression (PPD) screening. This study examines the prevalence of participation in PPD screening in the universal home-visiting programme in Denmark, in relation to migrant status and its association with acculturation factors, such as length of residence and age at migration.

**Methods:**

The sample consists of 77 694 births from 72 292 mothers (2015–18) that participated in the programme and were registered in the National Child Health Database. Lack of PPD screening using the Edinburgh Postpartum Depression Scale (EPDS) was examined in relation to migrant group and acculturation factors. We used Poisson regression with cluster robust standard errors to estimate crude and adjusted relative risk.

**Results:**

In total, 27.8% of Danish-born women and 54.7% of immigrant women lacked screening. Compared with Danish-born women, immigrant women in all groups were more likely to lack PPD screening (aRR ranging from 1.81 to 1.90). Women with low acculturation were more likely to lack screening. Women who migrated as adults [aRR = 1.27 (95% CI 1.16, 1.38)] and women who had resided in Demark for <5 years [aRR = 1.37 (95% CI 1.28, 1.46)] were more likely to lack screening.

**Conclusions:**

Immigrant women in Denmark, particularly recent immigrants, are at increased risk of not being screened for PPD using the EPDS. This can lead to under-recognition of PPD among immigrant women. More work is needed to understand how health visitors recognize the mental health needs of immigrant women who are not screened, and whether this gap results in reduced use of mental health services.

## Introduction

Postpartum depression (PPD) is a serious public health concern that affects between 10% and 15% of women globally.^1^ When untreated, it can have a negative, long-lasting impact on mothers’ wellbeing and children’s development.^2^ Early detection of signs of PPD is necessary to provide timely support.^3^ The systematic use of screening questionnaires, such as the Edinburgh Postpartum Depression Scale (EPDS) can increase the detection of women at risk for PPD, compared with routine clinical assessment.^4^^,^^5^ The EPDS is a highly used screening tool that has been translated into many languages and validated in a large range of populations.^6–8^ Early screening during preventive health care visits for children may reduce both the prevalence of PPD and depressive symptoms in women with PPD.^5^ Several countries, such as Sweden,^9^ Australia^10^ and Denmark recommend universal PPD screening. In Denmark, particularly, the EPDS has been used for almost two decades by health visitors (HVs) that care for infants and their families after birth.^8^

Lack of screening may hinder the identification of those at risk of PPD and subsequently have a negative impact on the delivery of timely mental healthcare. Moreover, the consequences may be more serious for immigrant women, particularly for refugees, given that they are at increased risk of developing PPD symptoms compared with the majority population in their destination country;^11^^,^^12^ partly explained by higher rates of social isolation, a history of stressful life events, low socio-economic status,^13^^,^^14^ discrimination and challenges in adjusting in the destination country.^15^^,^^16^

Qualitative research suggests that immigrant women experience challenges participating in PPD screening due to linguistic and cultural differences and stigma.^17^^,^^18^ From the perspective of healthcare providers, challenges using interpreters,^19^ and economic and time constraints during health encounters, may also influence the likelihood of screening.^20^ Despite all these concerns, only one study in Sweden has documented that immigrant women are less likely to be offered PPD screening using the EPDS when universal screening has been implemented in paediatric care,^21^ and no large studies have examined the characteristics of immigrant women who are more likely to remain unscreened.

Acculturation—the changes in culture and behaviours of an immigrant group as a result of interaction with the host community^22^—could influence participation in screening. Region of origin, length of residence and language proficiency in the new country have been used as proxies to measure acculturation.^23^^,^^24^ As such, becoming familiarized with the language and culture of the host country could facilitate participation in screenings. In addition, PPD screening is more widely documented in western countries, where women may share a similar understanding of PPD. In contrast, studies among different immigrant groups from non-western countries have reported cross-cultural differences in understandings of PPD symptoms that may influence participation.^19^^,^^25^ Moreover, migration status may also affect participation, given that particular experiences among refugees of flight, migration and reception in asylum centres could influence their trust in healthcare providers.^26^

To fill these gaps in our knowledge, this study aimed to determine the prevalence of PPD screening according to migrant status in the universal free home-visiting programme in Denmark, and to examine the association between proxies for acculturation and PPD screening among immigrant women. We hypothesized that immigrant women overall, and particularly those with lower acculturation, would be less likely to be screened for PPD compared with Danish-born women.

## Methods

### Study setting

Denmark has one of the oldest universal home-visiting programmes in Europe, in which child HVs offer free home visits to all families with infants, from birth to the child’s first birthday, with a participation rate of 95%.^27^ Following the national guidelines, HVs visit families five times a year on average, with the overall aim of promoting child health, screening for health and developmental problems, assessing parents’ mental health and facilitating access to specialized treatment.

Since 2002, HVs in a range of municipalities developed a clinical database—the Child Health Database (CHD)—in collaboration with the National Institute of Public Health, to store standardized data from their home visits. Each municipality in Denmark can choose whether to be part of the database. The database is based on HVs’ journal data. Data are obtained using a computerized healthcare journal, securing a uniform practice in data collection, in which data from each visit is registered. During the study period, two different data systems were used for registration.

EPDS has been used as a screening tool for PPD by HVs for the past 20 years. The national guidelines from the Danish Health Authorities ‘highly’ recommend the use of PPD screening tools at 8-weeks postpartum, but it is not mandatory. Therefore, this situation may create a variation between municipalities where each one can choose who is screened and how (i.e. screening for all vs. screening only when depression is suspected). Starting in 2015, HVs registered EPDS results and stored them in the CHD.

### Data sources and study population

We included mothers of all children born in the municipalities registering data (34 out of 98 municipalities) between 2015 and 2018, who were visited by a HV from birth to the child’s first birthday.

We identified 84 355 children born between 1 January 2015 and 31 December 2018 and their mothers (see Supplementary appendix for further details), registered in the CHD. To be included in the study, women had to have at least one visit registered (*n* = 78 255). We then excluded women who only received the first visit (*n* = 107), in which EPDS is not administered, those who died or whose children died (*n* = 21), who emigrated before the child’s first birthday (*n* = 240) and those with all data missing from the registries (*n* = 193) (Supplementary appendix figure S1). The final sample consisted of 77 694 births from 72 292 mothers, which accounts for one quarter of the total births in Denmark during the study period.

After obtaining each mother and father anonymized personal identification number, we linked it with a range of Danish population registries to obtain information on parental immigration status, sociodemographic variables and relevant information about the birth.

### Definition of variables

#### Migrant status

Based on the sociodemographic registry at Statistics Denmark, three groups of mothers were identified: Danish-born to Danish-born parents, Danish-born to immigrant parents (descendants) and immigrants (born outside Denmark to parents born outside Denmark). All immigrants had a valid residence permit at the time of giving birth to their child. Within the immigrants group, we distinguished between three subgroups: refugees, non-refugee immigrants from non-western countries and non-refugee immigrants from western countries. Refugee mothers were identified as individuals, who either had registered protection status or were reunified with a refugee family member (i.e. husband). There is no information on residence permit status prior to 1997, therefore, for the period 1980–97 refugees were identified based on year of immigration and country of origin.^28^ Immigrants that were not refugees (i.e. labour migrants, reunified to labour migrants and students) were then classified as originating from western and non-western countries, based on Statistics Denmark classification.^29^

#### EPDS screening completion

The outcome measure was lack of EPDS screening between 8-weeks postpartum and the child’s first birthday. National guidelines recommend screening at the 8-week visit but it could also be administered later.^30^ Lack of screening was identified when EPDS was not registered in the CHD.

#### Other variables

The child’s year of birth and municipality of residence was extracted from the CHD. Information on mothers’ date of birth, family composition and maternal education at child’s birth, and disposable household income, accounting for family size, and employment in the year prior to giving birth, were obtained from national registries. Maternal age was coded: <20 years, 20–29, 30–39 and ≥40 years. Family composition was coded as cohabitating with their partner/husband or not cohabitating. We calculated disposable household income quintiles. Education was coded: compulsory school, upper secondary and vocational school and college or university. Employment status in the year prior to birth was coded as employed (self-employed and employed with wages) and unemployed (women out of the labour market and women receiving unemployment benefits). In addition, information on gestational age and parity was obtained from the Medical Birth Registry. We then identified women that had experienced a premature birth (gestational age <250 days).

#### Acculturation-related predictors

For immigrant mothers, we identified their age at immigration and coded it as: <12 years, 12–17 and ≥18 and computed the length of residence in Denmark at the time of giving birth as: <5 years, 5–9 and ≥10 years of residence. We identified whether the mothers’ highest level of education was obtained in Denmark or abroad. In addition, we obtained the fathers’ country of origin. We then classified fathers based on migration status as Danish-born, descendant or immigrant. These variables were used as proxies for acculturation. Arriving as an adult, residing in Denmark for <5 years, having had education abroad, and not having a Danish-born partner were considered indicators of being less acculturated.^23^

### Statistical analysis

Mothers’ characteristics were compared across migration status and across those with and without screening, using chi-square tests. We used Poisson regression with cluster robust standard errors to account for non-independence within family clusters (multiple pregnancies per mother), to estimate the crude and adjusted relative risk (adjRR) of lacking PPD screening by migration status. This method is recommended for estimating risk ratios for common binary outcomes.^31^ We used the same procedure to examine the association between acculturation factors and lack of screening among immigrant women in a multivariable model. Crude risk ratios indicated the individual associations between each factor and a lack of screening. For adjusted prevalence ratios (RRs), all covariates were included in the model, regardless of statistical significance. Covariates of interest were defined *a priori* as characteristics known to be associated with PPD, differently distributed across migrant groups, and that conceivably could lead to differences in screening participation.^23^ These included maternal age, parity, pre-term birth, household income, employment in the year before giving birth, family composition and education. In addition, all models adjusted for municipality, cohort and data system (see Supplementary appendix section S1). Crude and adjRRs (RR and adjRRs) were estimated using the PROC GENMOD procedure in SAS, version 9.4.

In a supplementary analysis, we examined the possibility for interaction among exposure variables, estimated lack of screening among immigrants, stratified by acculturation factors, compared with Danish-born women and performed additional sensitivity analysis (see Supplementary appendix).

### Ethical approval

The CHD was approved by the Research & Innovation Organization at the University of Southern Denmark and complied with national regulations of data protection and consent. Data from the HVs’ records were stored at the National Institute of Public Health as per the Data Protection Legislation.^32^ Linkage with register-based data were administered by Statistics Denmark. Researchers did not have access to personal identification numbers.

## Results

### Study population

The final sample consisted of 3863 children born to refugee mothers, 5035 born to western immigrant mothers, 6725 born to non-western immigrant mothers, 3429 born to descendant mothers and 58 642 born from mothers in the majority population. Across the Danish-born, the descendants and the three migrant groups there were differences in maternal age, parity, education attainment, employment, family composition and family disposable income. Among immigrant women, refugees arrived at an earlier age and more often had resided in Denmark for at least 10 years. Non-refugees from western and non-western countries more often had a Danish-born partner compared with refugees (table 1).

**Table 1:** Sample characteristics by group based on total number of births

	Danish-born (*n* = 58 642)		Descendants (*n* = 3429)		Immigrant Western (*n* = 5035)		Immigrant non-Western (*n* = 6725)		Refugees (*n* = 3863)		χ^2^
Maternal characteristics	*N*	%	*N*	%	*N*	%	*N*	%	*N*	%	
Age at birth (median, SD)	31	4.9	28	4.89	31	4.78	32	4.92	30	5.76	
<20	361	0.6	52	1.5	30	0.6	27	0.4	77	2.0	1302.45^***^
20–30	24 116	41.1	2194	64.0	1653	32.8	2249	33.4	1722	44.6	
30–40	31 429	53.6	1115	32.5	3085	61.3	4082	60.7	1805	46.7	
>40	2736	4.7	68	2.0	266	5.3	367	5.5	259	6.7	
Parity											486.55^***^
Primiparous	29 090	49.6	1768	51.6	2611	51.9	2794	41.5	1277	33.1	
Multiparous	28 793	49.1	1627	47.4	2205	43.8	3621	53.8	2423	62.7	
Missing	759	1.3	34		219	4.3	310	4.6	163	4.2	
Pre-term birth											13.65^**^
Yes	2792	4.8	160	4.7	206	4.1	296	4.4	134	3.5	
No	55 115	94.0	3236	94.4	4615	91.7	6125	91.1	3570	92.4	
Missing	735	1.3	33		214	4.3	304	4.5	159	4.1	
Education											7397.75^***^
Compulsory	5582	9.5	713	20.8	292	5.8	1214	18.1	1402	36.3	
Upper secondary	19 425	33.1	1435	41.8	1252	24.9	1986	29.5	1134	29.4	
College or University	33 411	57.0	1204	35.1	3237	64.3	3050	45.4	893	23.1	
Missing	224	0.4	77	2.2	254	5.0	475	7.1	434	11.2	
Place of education											58.05^***^
From Denmark	–	–	–	–	2153	42.8	3232	48.1	1908	49.4	
From abroad	–	–	–	–	2674	53.1	3134	46.6	1765	45.7	
Missing	–	–	–	–	208	4.1	475	7.1	190	4.9	
Employment											8301.38^***^
Employed	52 683	89.8	2687	78.4	3909	77.6	4042	60.1	1437	37.2	
Unemployed	5741	9.8	708	20.6	789	15.7	2075	30.9	2044	52.9	
Missing	218	0.4	34	1.0	337	6.7	608	9.0	382	9.9	
Family composition											636.22^***^
Cohabitating	54 016	92.1	2864	83.5	4651	92.4	6004	89.3	3152	81.6	
Not cohabitating	4539	7.7	532	15.5	314	6.2	585	8.7	640	16.6	
Missing	87	0.1	33	1.0	70	1.4	136	2.0	71	1.8	
Family-adjusted disposable income (median/SD) in DKK	250 707	157 881	184 875	93 937	218 197	166 273	177 975	216 123	126 777	86 250	
High (1–3rd Q)	39 449	67.3	1315	38.3	2631	52.3	2313	34.4	673	17.4	6591.24^***^
Low (4–5th Q)	19 110	32.6	2081	60.7	2334	46.4	4276	63.6	3121	80.8	
Missing	83	0.1	33	1.0	70	1.4	136	2.0	69	1.8	
Migration characteristics											
Maternal length of residence					5	6.05	6	8.79	9	10	
(median, SD)											
<5 years	–	–	–	–	2194	43.6	2778	41.3	1659	42.9	1180.83^***^
5–9 years	–	–	–	–	1732	34.4	1431	21.3	294	7.6	
≥10 years	–	–	–	–	1108	22.0	2429	36.1	1901	49.2	
Maternal age at arrival					25	6.57	24	8.94	21	9.87	
(median, SD)											
<12	–	–	–	–	233	4.6	1044	15.5	1068	27.6	1385.41^***^
12–18 years	–	–	–	–	139	2.8	552	8.2	496	12.8	
≥18	–	–	–	–	4662	92.6	5042	75.0	2290	59.3	
Region											
Western-Nordic	–	–	–	–	1055	21.0	–	–	–	–	
Western-EU28	–	–	–	–	3765	74.8	–	–	–	–	
Western-non-EU	–	–	–	–	215	4.3	–	–	–	–	
East Europe-Central Asia	–	–	–	–	–	–	1866	27.7	498	12.9	
Middle East-North Africa	–	–	–	–	–	–	679	10.1	2107	54.5	
Sub-Saharan Africa	–	–	–	–	–	–	659	9.8	650	16.8	
South Asia-East pacific	–	–	–	–	–	–	3123	46.4	598	15.5	
South America-Caribbean	–	–	–	–	–	–	390	5.8	6	0.2	
Father migration status											49 921.79^***^
Danish-born	54 037	92.1	557	16.2	2007	39.9	1783	26.5	284	7.4	
Descendant	761	1.3	1402	40.9	107	2.1	676	10.1	113	2.9	
Immigrant	2703	4.6	1296	37.8	2832	56.2	4006	59.6	3259	84.4	
No information about father	1141	1.9	174	5.1	89	1.8	260	3.9	207	5.4	

Note: χ^2^, Chi-square.

** *P* < 0.01;

*** *P* < 0.001.

Women who lacked screening were more likely to be immigrants (33% vs. 13.7%), multiparous (53.2% vs. 48.0%) educated to a low level (15.3% vs. 10.1%), unemployed (21.1% vs. 11.4%), single (10.0% vs. 7.8%) and to have lower income (48.2% vs. 35.6%) compared to those screened (table 2). Among immigrant women, those not screened were more likely to have resided in Denmark for <5 years (54.2% vs. 28.3%), and to have arrived in Denmark as adults (83.9% vs. 68.2%), and were less likely to have a child with a Danish-born partner (19.0% vs. 34.6%) (*P* < 0.001) (table 2).

**Table 2:** Distribution (%) of predictors and covariates across screened and not screened groups

	Screened		Not screened		
	*N*	%	*N*	%	χ^2^
Total	51 814	66.7	25 880	33.3	
Group					4149.66^***^
Danish-born	42 356	81.7	16 286	62.9	
Descendants	2375	4.6	1054	4.1	
Immigrant Western	2513	4.9	2522	9.7	
Immigrant non-Western	3073	5.9	3652	14.1	
Refugees	1497	2.9	2366	9.1	
Age at birth					94.74^***^
<20	275	0.5	272	1.1	
20–30	21 566	41.6	10 368	40.1	
30–40	27 628	53.3	13 888	53.7	
>40	2344	4.5	1352	5.2	
Parity					298.74^***^
Primiparous	26 375	50.9	11 165	43.1	
Multiparous	24 896	48.0	13 773	53.2	
Pre-term birth					
Yes	2053	4.0	1535	5.9	
No	49 244	95.0	23 417	90.5	
Education					805.70^***^
Compulsory	5253	10.1	3950	15.3	
Upper secondary	17 355	33.5	7877	30.4	
College or University	28 799	55.6	12 996	50.2	
Employment					1479.89^***^
Employed	45 553	87.9	19 205	74.2	
Unemployed	5909	11.4	5448	21.1	
Family composition					122.29^***^
Cohabitating	47 740	92.1	22 947	88.7	
Not cohabitating	4022	7.8	2588	10.0	
Family-adjusted disposable income					1233.12^***^
High (1–3rd Q)	33 307	64.3	13 074	50.5	
Low (4–5th Q)	18 456	35.6	12 466	48.2	
Migration characteristics					
Maternal length of residence					561.65^***^
<5 years	2002	28.3	4629	54.2	
5–9 years	1730	24.4	1727	20.2	
≥10 years	3294	46.5	2144	25.1	
Maternal age at arrival					1161.13^***^
<12	1526	21.5	819	9.6	
12–18 years	672	9.5	515	6.0	
≥18	4828	68.2	7166	83.9	
Father migration status					569.77^***^
Danish-born	2453	34.6	1621	19.0	
Descendant	485	6.8	411	4.8	
Immigrant	3937	55.6	6160	72.1	

Note: Groups are based on number of births. Mothers can have more than one birth during the study period. Across variables, when the sum of *N* values do not sum up the total *N* of screened and not screened is dues to missing data on that particular variable. χ^2^, Chi-square.

*** *P* < 0.001.

### Lack of screening by migration status compared to Danish-born women

The prevalence of lack of screening for Danish-born women was 27.8%, compared to 30.7% for descendants, 50.1% for western immigrants, 54.3% for non-western immigrants and 61.1% for refugees. In simple and multivariable Poisson regression models, a significant association was seen between migration status and lack of screening. The fully adjusted relative risk (aRR) for lack of screening was 1.82 for western immigrants (95% CI 1.75, 1.88), 1.90 for non-western immigrants (95% CI 1.84, 1.96) and 1.81 for refugees (95% CI 1.74, 1.87) compared to Danish-born women. In addition, descendants of migrants also had a higher relative risk [aRR 1.27 (95% CI 1.20, 1.33)] of lack of screening compared to Danish-born women (table 3).

**Table 3:** Crude and adjusted risk ratio for lack of screening vs. screening

			Model 1		Model 2		Model 3	
	Crude relative risk ratio	95% confidence interval	Adjusted relative risk ratio	95% confidence interval	Adjusted relative risk ratio	95% confidence interval	Adjusted relative risk ratio	95% confidence interval
Group								
Danish-born	ref.		ref.		ref.		ref.	
Descendants	1.12	1.06–1.18	1.33	1.26–1.40	1.33	1.26–1.40	1.27	1.20–1.33
Immigrant Western	1.82	1.76–1.88	1.86	1.81–1.93	1.88	1.82–1.95	1.82	1.75–1.88
Immigrant non-Western	1.98	1.92–2.03	2.10	2.04–2.16	2.09	2.03–2.15	1.90	1.84–1.96
Refugees	2.22	2.15–2.28	2.20	2.13–2.27	2.14	2.08–2.21	1.81	1.74–1.87
Age at birth								
<20	1.53	1.40–1.67			1.54	1.40–1.69	1.33	1.21–1.46
20–30	ref.				ref.		ref.	
30–40	1.11	1.07–1.14			0.99	0.97–1.01	1.04	1.02–1.07
>40	1.28	1.21–1.37			1.07	1.02–1.12	1.12	1.07–1.17
Parity								
Primiparous	ref.				ref.		ref.	
Multiparous	1.37	1.13–1.67			1.17	1.15–1.19	1.14	1.12–1.17
Pre-term birth								
No	ref.				ref.		ref.	
Yes		1.31–1.46			1.31	1.26–1.36	1.30	1.25–1.35
Education								
Compulsory	ref.						ref.	
Upper secondary	1.29	1.14–1.47					1.06	1.03–1.09
College or University	2.20	1.91–2.52					1.14	1.05–1.24
Employment								
Employed	ref.						ref.	
Unemployed	1.79	1.54–2.08					1.17	1.14–1.20
Family composition								
Cohabitating	ref.						ref.	
Not cohabitating	1.17	1.07–1.26					1.06	1.02–1.10
Family-adjusted disposable income								
High (1–3rd)	ref.						ref.	
Low (4–5th Q)	1.5	1.40–1.60					1.13	1.10–1.16

### Acculturation proxies and lack of screening among immigrant women

**Figure 1:**
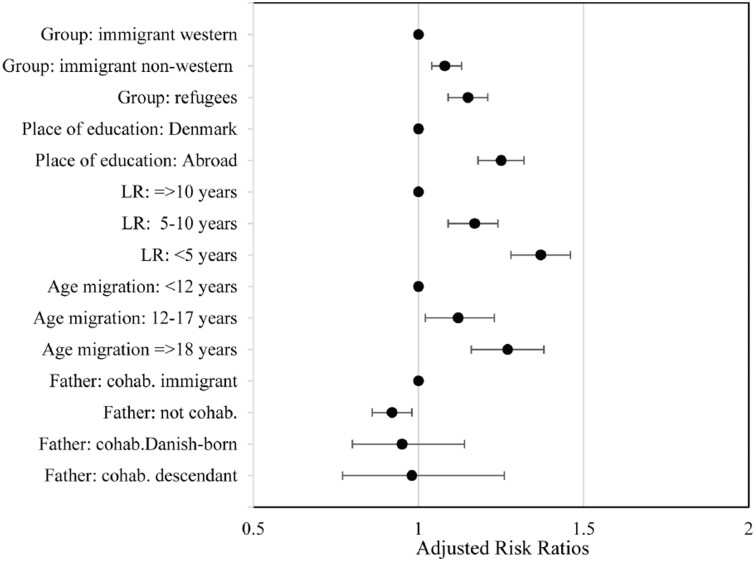
adjRR for lack of screening within migrants

Among immigrant women, a significant association was seen between all acculturation-related factors and lack of screening (figure 1 and Supplementary appendix table S1). Refugee and non-western immigrants had increased adjRR of lack of screening [aRR 1.15 (95% CI 1.09, 1.21); aRR 1.08 (95% CI 1.04, 1.13)], compared to western immigrants. No differences were seen between refugee and non-western immigrants. Immigrant women who had resided in Denmark for <5 years had the highest relative risk of lacking screening [aRR 1.37 (95% CI 1.28, 1.46)] compared to women that lived in Denmark for more than 10 years, regardless of their migration status. For age on arrival in Denmark, we found that women arriving as adults [aRR 1.27 (95% CI 1.15, 1.42)], and those who arrived between 12 and 18 years of age [aRR 1.20 (95% CI 1.16, 1.38)] were at increased risk of not being screened, compared to those that arrived before the age of 12. Women who had studied abroad also had increased relative risk [aRR 1.25 (95% CI 1.18–1.32)] compared to women who had studied in Denmark. There were no statistically significant interactions between migration status and acculturation variables.

## Discussion

Overall, lack of screening was present among all groups. In total, 27.8% of Danish-born women lacked screening, which is comparable to prior studies in other countries.^21^ However, the results show a greater risk of lack of screening for immigrant women. Compared to Danish-born women, non-western immigrant women were 90% more likely to lack screening, followed by western immigrants (82%) and refugees (81%). Within immigrants, both refugee and non-western women were 15% and 9% more likely than western immigrants to lack screening, independent of other acculturation and sociodemographic factors. Generally, all factors indicating lower acculturation, such as shorter length of residence, older age at migration and having studied abroad were independently associated with increased risk of lack of screening for all migrant groups. The strongest predictor for lack of screening was length of residence, with migrant women who had resided in Denmark for <5 years being 37% more likely to lack screening compared with women who had resided in Denmark for more than 10 years. Nonetheless, even the more acculturated groups that arrived in Denmark before the age of 12, or resided in the country for more than 10 years, were more likely to lack screening compared with Danish-born women (Supplementary appendix table S2). In addition, descendants were also at higher risk of lacking screening compared with Danish-born women.

Our findings showing lower overall screening among immigrants are in line with previous European studies on participation in other types of health screening among immigrants^33^ and may reflect challenges in PPD screening practices reported in prior qualitative studies.^20^ In our study, screening participation was generally similar in refugee and non-refugee non-western immigrants, suggesting that despite the fact that refugee women may be at increased risk of PPD, they experience similar barriers to participating in PPD screening as non-refugee non-western immigrants. These two groups had lower participation than western migrants, which was not fully explained by different acculturation and sociodemographic patterns, suggesting that other factors, not included in this study, related to cross-cultural understandings of PPD symptoms and healthcare system barriers may influence participation.

Multiple mechanisms related to cultural and linguistic differences may influence lack of screening. First, results showing that women who resided in Denmark for <5 years were the group with the highest risk of lack of screening suggest that Danish language proficiency and limited knowledge about the offers and structure of the Danish health care system may contribute to lack of screening.^34^ Recent migrants may have less knowledge of the Danish language and therefore, if translated versions or interpreters are lacking, nurses may appropriately choose not to screen. In addition, even if interpreters are available, some women might restrain from discussing their mental status in their presence.^21^ However, immigrant women who arrived in Denmark before the age of 12, and therefore attended school in Denmark, also showed a 41% increased risk of lack of screening compared with Danish-born women (Supplementary appendix table S2). Therefore, other factors besides language barriers may explain such differences. For example, stigma around PPD, lack of information about PPD, or different understanding of mental health symptoms, could lead some immigrant women to decline participation.^20^^,^^35^ Moreover, cultural misconceptions, lack of time to use interpreters, or a need to prioritize other family issues, could influence the HV decision of whether to offer screening or not. Further research should explore these different explanations and understand the perspectives of both immigrant women and HVs engaged in screening for PPD.

Our results undoubtedly point out an important gap in practice that leads to an additional question: if immigrant women do not participate in screening for PPD, what practices are in place to identify their potential mental health concerns after birth? Research in Sweden suggests that nurses use their tacit knowledge and intuition to identify women in need.^36^ Yet, this may not be enough to identify immigrant women at risk of PPD and may be influenced by the nurses’ cultural competence. Some scholars have questioned the use of tools such EPDS among culturally and linguistically diverse populations,^37^ suggesting that it may lack cultural appropriateness. However, recent research suggests that using a computerized translated version, together with a psychosocial screening in midwives practice, is in general well accepted among refugee women and healthcare providers.^38^ More research is needed to build up evidence about screening practices and tools that can overcome potential barriers to identifying immigrant women in need of mental health support after birth.^33^ A focus on postpartum mental health literacy to enhance participation in screening is needed.^37^^,^^39^

To our knowledge, this is the first large quantitative study to examine differences in PPD screening rates between migrant and non-migrant women, and to examine the role of acculturation factors when screening is recommended in national guidelines. However, several limitations must be considered. Despite results being based on a large sample that represents one quarter of births in Denmark from 2015 to 2018, they only represent one-third of all Danish municipalities. Therefore, caution must be used when generalizing findings to all Danish municipalities and countries beyond the Nordic welfare states. Nonetheless, barriers to health screening participation among migrants have been documented in others countries.^20^ Thus, inequalities in PPD screening could also be expected in other contexts if these are not actively prevented.

In addition, this study was not conducted as part of a controlled implementation of EPDS; it was based on routine data collected under real world conditions, which adds some methodological challenges. Despite EPDS being registered every time it was administered, we lacked data on the mode of administration (self-administered, read by the nurse, with an interpreter) and reasons for not screening. Thus, we could not identify whether women refused to participate or the screening was not offered. In addition, despite identifying different proxies for acculturation, we lacked a measure for language proficiency or need for an interpreter, which has been described as one of the main barriers to implementation of PPD screening.^19^ Moreover, despite interpretation services being available to healthcare providers in Denmark, we lacked data on interpreters’ availability at the time of the home visit. Therefore, we could not know if the lack of screening was explained by the lack of reliable interpreters.

Despite these limitations, we identified several acculturation factors and examined their association with lack of screening, while adjusting for municipality, cohort, journal system and sociodemographic factors. These factors inform potential reasons for lack of screening and help to identify groups that may be at increased risk of lack of PPD screening. In addition, our extensive sensitivity analysis (Supplementary appendix sections S6–S8) showed no changes in results, suggesting robustness of findings.

In conclusion, this study documents a greater risk of lack of PPD screening for immigrant women, particularly for those less acculturated. Lack of screening is indicating inequities in real world home-visiting settings that may result in larger unmeet mental health needs among immigrant women. Policymakers and clinicians implementing universal PPD screenings should be aware of such potential inequality and both examine and tackle barriers to effectively screen and recognize the mental health needs of immigrant women.

## Supplementary data

Supplementary data are available at *EURPUB* online.

## Supplementary Material

ckab197_Supplementary_Data
