# Supplementary material for: Disparities in postpartum depression screening participation between immigrant and Danish-born women
Source: Eur J Public Health. 2021 Dec 2;32(1):41–8. doi: 10.1093/eurpub/ckab197 (PMC9090168; doi:10.1093/eurpub/ckab197)
Supplement: ckab197_Supplementary_Data [file ckab197_supplementary_data.docx]

**Appendix**

**Supplementary material**

In these appendix section we expand upon relevant methodological information that could not be included in the manuscript due to space limitations. We also present several sensitivity analysis.

1. **Data collection systems:**

Journal data was entered using two different journal systems called TMS and NOVAX. The systems are different manly because while the TMS system (only) has a considerable number of mandatory data common for all users, the NOVAX system is flexible and only includes a small common core of data. However, both journals register EPDS data, therefore not affecting our study. Regardless, we adjust all our analysis for the journal system used.

1. **Sample selection:**

Figure S1 shows the sample selection flow:

84355

78255

No visits registered

N=6100 (7.2%)

Only visit A

N=107

Figure S1. Sample selection

78148

Mother or child died 0-1 year

N=21

78127

77887

77694 children

Missing all registry data

N=193

Emigrated 0-1 year

N=240

In preparing the data for the present study, we detected an informatics glitch within the TMS journal that affected EPDS data. For mothers that had two children during the study period, the information registered about EPDS for the second child could not be exported into the National Child database. Therefore, when mothers in TMS had two children during the study period (2015-2018), only data from the first birth was included (n=36885, 75%). Any birth after the first one was excluded (n=12702, 25%). These children are not included in the initial sample of 84,355 births.

1. **Differences between mothers’ children and their mothers included in the study and those excluded due not having a registered visit.**

In the study selection, we excluded cases that were registered in the database but had no visits registered. When visits are not registered is because the family refused to receive the visit or the family moved to a municipality that does not give their data to the National Child Health database. Here we report differences between mothers excluded due to not having visits registered and those included in the study.

We observed differences between mothers and children that were excluded and those that remained in the study. There was a larger percentage of non-refugee immigrant mothers in the excluded group than in the final sample (26.44% in the excluded groups vs. 15,14% in the final sample) and this difference was significant (p<.001). The excluded group was more likely to have had a preterm birth (6.30% vs.4.61%), less likely to be nulliparous (43.84% vs. 48.32%), and less likely to have obtained university education (45% vs. 53.79%), and be single (12.11% vs 8.51%) (p<.001). No significant differences were observed in other variables.

1. **To supplement Figure 1, we include the table with all the risk ratios and CI.**

| Table S1. Crude and Adjusted Relative risk for lack of screening within migrants (N=15623) | | | | | |
| --- | --- | --- | --- | --- | --- |
|  | Crudde Relative risk ratio | **95% confidence interval** |  | Fully Adjusted Relative risk ratio | **95% confidence interval** |
|  |  |  |  |  |  |
| **Migrant status** |  |  |  |  |  |
| Non-Refugee Western | Referent |  |  | Referent |  |
| Non-Refugee Non-Western | 1.08 | 1.05-1.12 |  | 1.08 | 1.04-1.13 |
| Refugee | 1.22 | 1.17-1.26 |  | 1.15 | 1.09-1.21 |
| **Place of education** |  |  |  |  |  |
| Denmark | Referent |  |  | Referent |  |
| Abroad | 1.58 | 1.53-1.63 |  | 1,25 | 1.18-1.32 |
| **Lentgh of time** |  |  |  |  |  |
| More than 10 years | Referent |  |  | Referent |  |
| Between 5 and 10 | 1.27 | 1.21-1.33 |  | 1.17 | 1.09-1.24 |
| Less than 5 years | 1.77 | 1.71-1.84 |  | 1.37 | 1.28-1.46 |
| **Age at arrival** |  |  |  |  |  |
| Less than 12 | Referent |  |  | Referent |  |
| From 12 to 17 | 1.25 | 1.44-1.36 |  | 1.12 | 1.02-1.23 |
| 18 or more | 1.72 | 1.62-1.82 |  | 1.27 | 1.16-1.38 |
| **Father migration** |  |  |  |  |  |
| Immigrant | Referent |  |  | Referent |  |
| Danish-born | 0.79 | 0.66-0.93 |  | 0.95 | 0.80-1.14 |
| Descendant | 0.78 | 0.62-0.98 |  | 0.98 | 0.77-1.26 |
| Not cohabitating | 0.90 | 0.84-0.96 |  | 0.92 | 0.86-0.98 |
| *Note.* The first adjusted model includes municipality, cohort, and data system. The fully adjusted models adds age at birth, parity, preterm birth, maternal education, employment, civil status, and family disposable income. Fully adjusted models N=13260 dues to missing data on covariates | | | | | |

1. **Risk ration of lack of screening among immigrant women by acculturation factors compared to Danish-born women**

To inform our research question regarding how acculturation factors influenced EPDS screening, we compared the group of immigrant women stratified by each acculturation factor independently to Danish-born women. These analyses would allow us to examine whether the more acculturated immigrant women had different screening rates than Danish-born women. In fully adjusted models, immigrant women who had resided in Denmark for less than 5 years had the highest relative risk of lack of screening (aRR 2.36 [95%CI 2.29,2.42]) compared to Danish-born women, followed by those who resided between 5 and 10 years (aRR 1.82 [95%CI 1.76,1.89]) and those that resided for more than 10 years (aRR 1.41 [95%CI 1.36,1.47). Women arriving as adults had the highest risk of lack of screening (aRR 2.02 [95%CI 1.98,2.07) compared to Danish-born women, followed by those who arrived between 12 and 18 years of age (aRR 1.50 [95%CI 1.40,1.60), and those who arrived before age 12 (aRR 1.29 [95%CI 1.22,1.37). We found similar patterns with place of education and father’s origin (Table S2). Therefore, even the more acculturated groups were more likely to remain unscreened when compared to Danish-born women.

| Table S2. Relative risk of lack of screening among immigrants compared to Danish-born women stratified by acculturation factors | | |
| --- | --- | --- |
|  | Adj. RR | **95% CI** |
| Danish-born | Ref. |  |
| Immigrant education in DK | 1.54 | 1.49-1.58 |
| Immigrant education abroad | 2.24 | 2.19-2.31 |
|  |  |  |
| Danish-born | Ref. |  |
| Immigrant More than 10 years | 1.41 | 1.36-1.47 |
| Immigrant Between 5 and 10 | 1.82 | 1.76-1.89 |
| Immigrant Less than 5 years | 2.36 | 2.29-2.42 |
|  |  |  |
| Danish-born | Ref. |  |
| Immigrant arrived < 12 years | 1.29 | 1.22-1.37 |
| Immigrant arrived between 12 to 18 | 1.50 | 1.40-1.60 |
| Immigrant arrived > 18 | 2.02 | 1.98-2.07 |
|  |  |  |
| Danish-born | Ref. |  |
| Immigrant with Danish partner | 1.38 | 1.33-1.44 |
| Immigrant with Descendant partner | 1.76 | 1.62-1.91 |
| Immigrant with Immigrant partner | 1.81 | 1.77-1.86 |
| *Note*. For each acculturation factor, we have estimated separately the crude and adjusted relative risk of lack of screening. The adjusted models include municipality, cohort, and data system; age at birth, parity, preterm birth, maternal education, employment, civil status, and family disposable income. N=74545 due to missing data in covariates | | |

1. **Sensitivity analysis 1: We calculated the Risk ratio for lack of EPDS screening including all mothers and children excluded from the final sample.**

Overall, the ratio of women lacking screening increased for all groups was slightly higher but we found similar differences between immigrant groups and Danish-born and similar acculturation determinants of lack of EPDS. The percentage of women lacking EPDS screening was 31.74% in Danes compared to 35.54%% in Descendants, 55.96%% in western immigrants, 60.31%% in non-western immigrants, and 64.15% in Refugees. In simple and multivariable Poisson regression models, similar significant association was seen between population groups and lack of screening. The relative risk [RR] for lack of screening was 1.78 for western immigrants (95% CI 1.73,1.82), 1.92 for non-western immigrants (95% CI 1.87,1.95), and 2.03 for refugees (95% CI 1.97,2.08) compared to Danish-born women. Similar acculturation determinants of lack of EPDS screening were found. Women who migrated as adults, who had resided in Denmark for less than 5 years, and had education from abroad had increased risk of lack of screening compared to Danes and within migrants.

1. **Sensitivity analysis 2**: **We calculated risk ratio including only children with a registered visit at 8 weeks post birth (n=73222).**

We perform such analysis because the national guidelines recommend to screen with the EPDS in the 8 week visit, despite the screening could also be done at a later visit. Similar determinants of lack of EPDS screening were found. The percentage of women lacking EPDS screening was 24.96% in Danes compared to 27.48% in Descendants, 47.09% in western immigrants, 51.31% in non-western immigrants, and 59.25% in Refugees. In simple and multivariable Poisson regression models, significant association was seen between population groups and lack of screening. The fully adjusted relative risk [aRR] for lack of screening was 1.92 for western immigrants (95% CI 1.85,1.98), 2.00 for non-western immigrants (95% CI 1.95,2.07), and 1.93 for refugees (95% CI 1.86,2.01) compared to Danish-born women. Similar acculturation determinants of lack of EPDS screening were found. Women who migrated as adults, who had resided in Denmark for less than 5 years, and had education from abroad had increased risk of lack of screening compared to Danes and within migrants.

1. **Moderation analysis:**

In simple and multivariable Poisson regression models we examine potential interaction among exposure variables. In the model testing the effect of migrant status (table 3) all the following interaction were non-significant 1) migrant status X employment, 2) migrant status X income, 3) migrant status X civil status, 4) migrant status X education. We also tested potential interactions between acculturation when examining the association between acculturation factors and lack of screening (figure 2). Tested interactions were non-significant: migrant status X age at arrival, migrant status X length of time.
